# Rapid lateral flow immunoassay for fluorescence detection of canine distemper virus (CDV)

**DOI:** 10.3389/fvets.2024.1413420

**Published:** 2024-06-11

**Authors:** Zhigang Cao, Li Yi, Xiangnan Liu, Jinyuan Shang, Yuening Cheng, Erkai Feng, Xingyu Liu, Yuping Fan, Xiaoliang Hu, Wenlong Cai, Feng Cong, Shipeng Cheng

**Affiliations:** ^1^Institute of Special Animal and Plant Sciences, CAAS, Changchun, Jilin, China; ^2^Department of Infectious Diseases and Public Health, Jockey Club College of Veterinary Medicine and Life Sciences, City University of Hong Kong, Kowloon, Hong Kong SAR, China; ^3^Guangdong Laboratory Animals Monitoring Institute and Guangdong Provincial Key Laboratory of Laboratory Animals, Guangzhou, China; ^4^Faculty of Agriculture, Forestry and Food Engineering, Yibin University, Yibin, China

**Keywords:** CDV, FLFA, fluorescent, microspheres, detection

## Abstract

Canine distemper virus (CDV) is a highly contagious and potentially lethal virus that affects dogs and other members of the Canidae family, including wolves, foxes, and coyotes. Here, we present a fluorescent lateral flow immunoassay (FLFA) platform for the detection of CDV, which utilizes fluorescent microspheres - fusion protein monoclonal antibody (mAb)-labeled monoclonal antibody. The assay detected CDV within 5 min, with a detection limit threshold of 3 × 10^2^ TCID_50_/mL. Notably, the assay demonstrated no cross-reactivity with canine parvovirus, canine coronavirus, canine adenovirus, feline calicivirus, feline herpesvirus, or feline parvovirus. Field and clinical applicability of the assay was evaluated using 63 field samples, including 30 canine fecal samples, 18 swab samples, and 15 blood samples. The coincidence rate between the detection results of clinical samples obtained through FLFA and reverse transcription polymerase chain reaction (RT-PCR) was 96.83%. Thus, this assay offers a significant advancement for the rapid diagnosis of CDV at the point of care.

## Introduction

Canine distemper virus (CDV) is an enveloped, non-segmented, negative-stranded, negative-sense RNA virus classified in the *Morbillivirus* genus of the *Paramyxoviridae* family. CDV infections can lead to systemic disease involving the central nervous system as well as the respiratory and gastrointestinal tracts (1). The CDV genome spans 15,690 nucleotides in length and consists of six motifs encoding six structural proteins, including the nucleocapsid (N), phosphoprotein (P), matrix (M), fusion (F), hemagglutinin (H), and large (L) proteins.

Canine distemper (CD) is a multisystemic disease impacting a wide variety of animal families, including members of *Canidae*, *Felidae*, *Mustelidae*, *Procyonidae*, *Ursidae*, *Phocidae*, *Viverridae*, *Hyaenidae*, *Ailuridae*, *Mephitidae*, *Muridae*, *Cricetidae*, and *Cercopithecidae* (2). The pathogenesis and progression of the disease manifest through biphasic fever, systemic involvement of the respiratory and gastrointestinal systems, and neurological symptoms. Consequently, the development of rapid and accurate detection methods for CDV is crucial for clinical diagnosis.

While lateral flow immunoassays are widely used for on-site detection because of rapid time-to-result readout and ease of operation (3–5), it is a challenge to generate sufficient signal intensity and a high degree of sensitivity (6, 7). Because the optical signal can be amplified remarkably by doping microspheres. Fluorescent lateral flow immunoassay (FLFA) has a great advantage in terms of sensitivity (8, 9). Techniques for virus isolation, specific antibody detection and immunochromatography assay have been established for the diagnosis and management of CDV (10, 11). However, there remains a pressing need for rapid and accuracy diagnostic methods, including point-of-care testing, for effective disease management, especially in wildlife, where signs and symptoms may not be readily apparent. In the current study, we introduce a fluorescent lateral flow immunoassay (FLFA) platform for the detection of CDV. This method is sensitive and specific for CDV determination in both clinical settings and various research contexts.

## Materials and methods

### Ethics statement

No animals were sacrificed specifically for this study. Samples of feces, blood, and swab were collected by the veterinary hospital in Changchun and Guangzhou. The owner of each dog took the initiative to send the sick animal to the veterinary hospital for treatment, and the samples were collected and diagnosis by the laboratory.

### Reagents and antibodies

The fusion protein monoclonal antibodies (F-mAb) for CDV were purchased from HyText Co., Ltd. An immunochromatographic strip, including sample pad, absorbent pad, PVC pad, and nitrocellulose (NC) membrane, was purchased from Jiening Bio Co., Ltd. The fluorescence microsphere, 1-ethyl-3-(3-dimethylaminopropyl) carbodiimide (EDC), N-hydroxysuc-cini-mide, bovine serum albumin (BSA) and 2-(N-morpholino) ethanesulfonic acid (MES) were purchased from Sigma (St. Louis, MO, United States).

### Construction of fluorescent microspheres-monoclonal antibody (mAb)-labeled probe

A fluorescent microspheres-monoclonal antibody (mAb)-labeled probe was prepared as follows steps. Briefly, microsphere was suspended in 1 mL 0.01 M sodium dihydrogen phosphate solution (NaH_2_PO_4_) with pH 6.0. After centrifugal at 8,000 × g for 2 min, 1 mL of activation solution (10 mg EDC and 6 mg NHS dissolved in 1 mL of 0.01 M NaH_2_PO_4_) was resuspended the microspheres for 15 min. After that the microspheres were activated. Then, 100 μL 50 mM pH 5.0 MES were added into the activated microspheres and coupled with 100 μg ~ 500 μg antibodies as labeled antibodies for incubation 2 h, then blocked with 1 mL blocking solution (pH 7.4 0.01 M PBS, 1% BSA). The fluorescent microspheres-monoclonal antibody (mAb)-labeled probe was finally resuspended in 1 mL of storage solution (pH 7.4 0.01 M PBS, 1% BSA and 0.1% sodium azide) and stored at 4°C for further use. All experiments were performed in triplicate.

### Preparation of standard CDV fusion antigen

Recombinant CDV fusion antigen samples of various concentration (10, 20, 30, 40 and 50 ng/μL) were prepared by diluting an appropriate amount of the antigen with diluent buffer (PBS containing 1% bovine serum albumin) into 100 μL in PBS. These standard antigen samples were measured in triplicate by the CDV fusion assay.

### Fabrication of F lateral flow strip

The immunochromatographic test strip was composed of a sample pad, a conjugate pad, a NC membrane, and an absorption pad. The F-mAb-BSA and goat anti-mouse immunoglobulin G (IgG) were spotted on the NC membrane using the BioDot XYZ platform (California, United States) as the test (T line) and control lines (C line), respectively. The prepared NC membranes were dried at 37°C for 12 h. The absorption and conjugate pads were used without treatment. All experiments were performed in triplicate.

### Sensitivity of F-FLFA

The CDV reference strain (Snyder Hill, Accession No. GU138403) was cultured in VERO cell stored in our laboratory. Serial 10-fold dilutions of CDV (1 × 10^3^ TCID_50_/mL to 1 × 10^1^ TCID_50_/mL) were used to assess the detection limit of the F-FLFA.

### Specificity of F-FLFA

The specificity of F-FLFA was assessed using CDV, canine parvovirus (CPV), canine coronavirus (CCoV), canine adenovirus (CAV), feline calicivirus (FCV), feline herpesvirus (FHV), and feline parvovirus (FPV). Sterile phosphate-buffered saline (PBS, 0.01 M, pH 7.4) was used as a negative control for RNA and DNA extraction (12). In brief, Viral RNA was extracted using TRIzol reagent (TaKaRa Biotechnology, Dalian, China) and viral DNA was extracted using the DNA Mini Kit (50) (Omega Bio-tek, Norcross, GA, United States), following the manufacturers’ protocols. The F-FLFA reaction was carried out under optimized conditions. All samples were tested in duplicate.

### Testing of field samples

In total, 63 field samples collected by pet hospitals were derived from different individuals and agreed with owers, including 30 canine fecal samples, 18 oral and nasal mixed swab samples, and 15 blood samples, were tested using F-FLFA and then compared to RT-PCR based on the P gene (13) to evaluate for any nonspecific amplification. The nucleic acid of CDV served as a positive control in these tests.

### Statistical analysis

GraphPad Prism software version 8.0 (GraphPad, Inc., La Jolla, CA, United States) was used to determine the means and SDs. The Coincidence rate (CR) = (positive amount + negative amount) / total amount × 100%.

## Results

### F-FLFA mechanism

**Figure 1:**
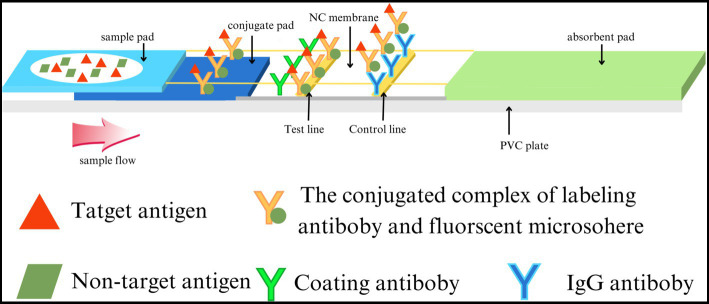
Structure and principle of the F-FLFA test strip.

**Figure 2:**
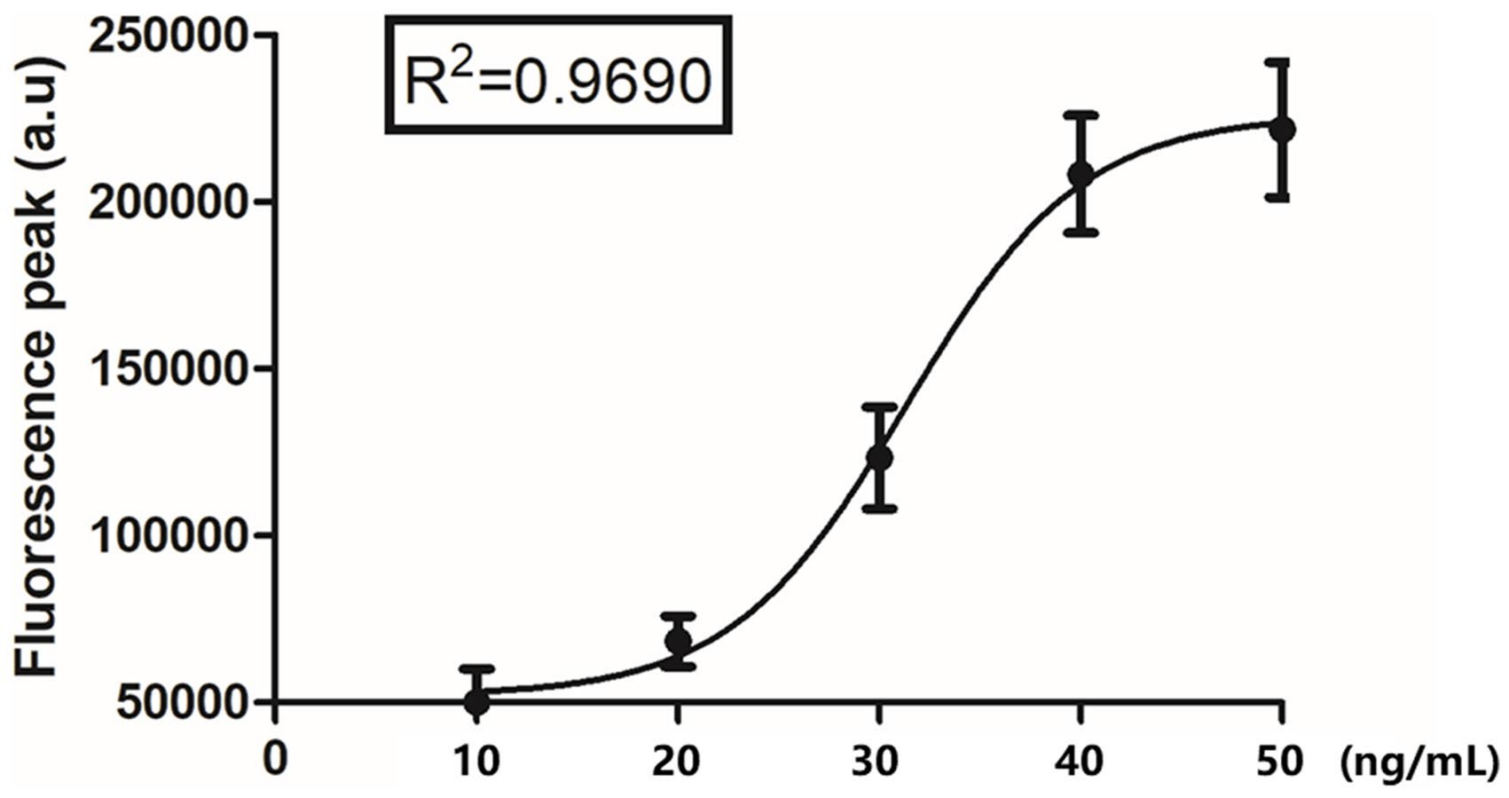
The relationship between the detected fluorescence intensity and different fusion concentration (10 ng/mL, 20 ng/mL, 30 ng/mL, 40 ng/mL, and 50 ng/mL).

Mouse anti-CDV F-mAb was prefixed on the test line (T) of the lateral flow strip, while the control line (C) was coated with goat anti-rabbit IgG polyclonal antibodies. The reaction tube contained fluorescent microspheres-mAb and rabbit IgG. Initially, the supernatant of tissue homogenate, serum, and anticoagulation blood samples were tenfold diluted with dilution buffer for lysing and the releasing viral particles were bound to fluorescent microspheres -mAb. The resulting complex flowed along the sample pad and NC membrane towards the absorbent pad under capillary force. As the complex passed the T line, it was captured by the F-mAb, leading to the generation of a fluorescent signal. At the C line, fluorescent microspheres -bound rabbit IgG was captured by the goat anti-rabbit IgG, resulting in a control signal (Figure 1 and Supplementary Figure S1). For F-FIFA assay, the linearity range (*R*^2^ = 0.9690) was extended up to 50 ng/mL (Figure 2).

### Specificity and sensitivity of F-FLFA

**Figure 3:**
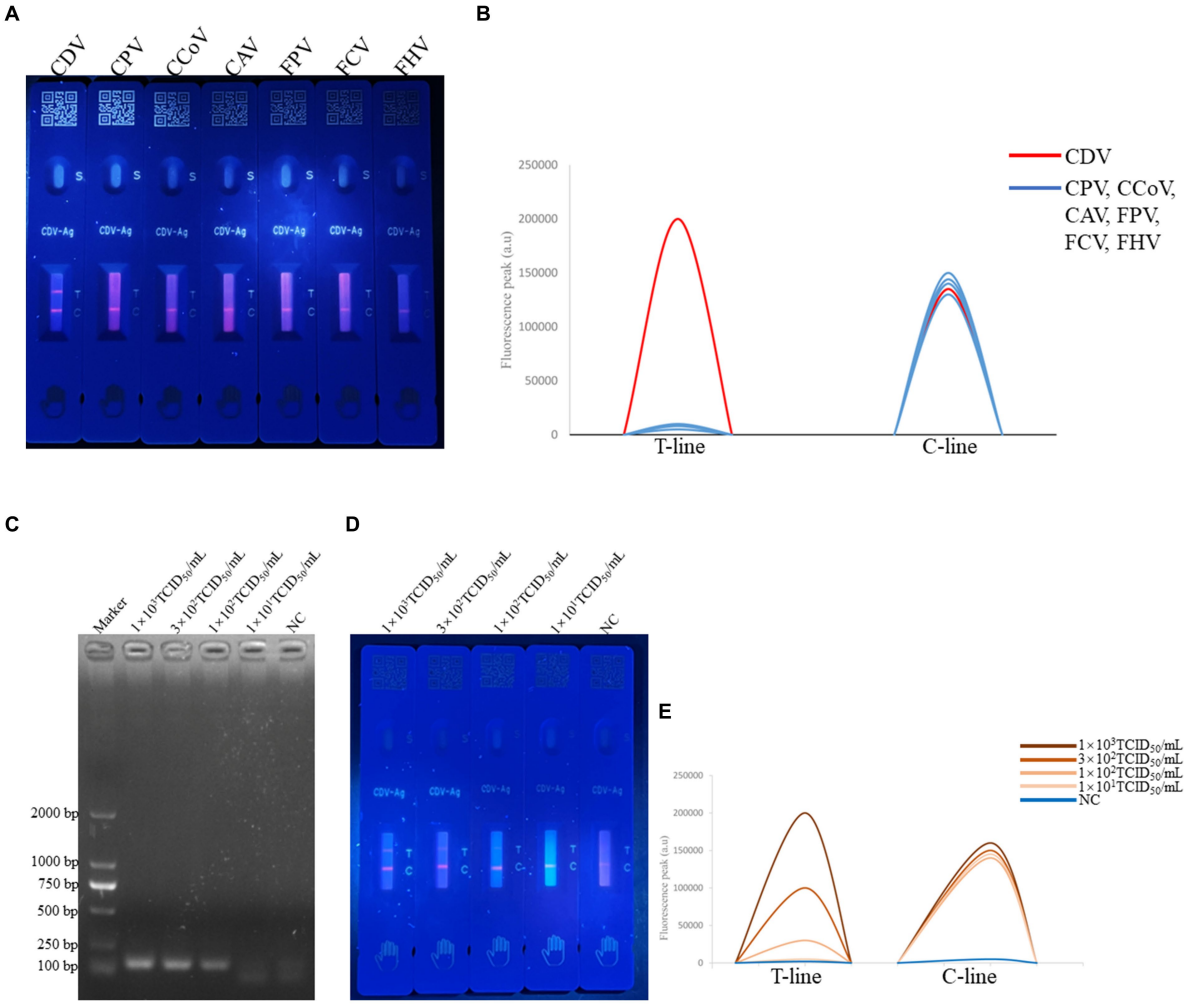
Specificity of F-FLFA assay for the detection of CDV, CPV, CCoV, CAV, FPV, FCV and FHV **(A)** and readout of fluorescence intensity of test and control lines **(B)**; sensitivity RT-PCR assay based on the P gene for the detection of CDV, PCR results confirmed by 2% agarose gel electrophoresis. The amplicon size of PCR production is 116 bp. Lane 1, DL-2000 DNA marker (TAKARA, Dalian, China). Lanes 2 to 5, corresponding to the concentrations of 1 × 10^3^ TCID_50_/mL, 3 × 10^2^TCID_50_/mL, 1 × 10^2^TCID_50_/mL, 1 × 10^1^TCID_50_/mL. Lanes 6, NC indicates a negative control **(C)** and sensitivity F-FLFA assay for the detection of CDV, Lanes 1 to 4, corresponding to the concentrations of 1 × 10^3^ TCID_50_/mL, 3 × 10^2^TCID_50_/mL, 1 × 10^2^TCID_50_/mL, 1 × 10^1^TCID_50_/mL. Lanes 5, NC indicates a negative control **(D)** and readout of fluorescence intensity of test and control lines **(E)**.

Seven relevant canine and feline pathogens, including that of CPV, CCoV, CAV, FCV, FHV, FPV, and CDV, were tested in this study. As shown in Figure 3, only CDV-positive samples presented obvious immunocomplexes, with the six other pathogens and control (PBS buffer) displaying negative reactions, thus illustrating high specificity of the established F-FLFA (Figures 3A,B).

To evaluate the sensitivity of F-FLFA, CDV was serially diluted to concentrations ranging from 10^3^ to 10^1^ TCID_50_/mL. As shown in Figures 3C–E, the limits of detection (LODs) for CDV by RT-PCR and F-FLFA were 10^1^ TCID_50_/mL and 3 × 10^2^ TCID_50_/mL, respectively. Thus, these results suggest that the LOD of F-FLFA is comparable to that of RT-PCR.

### Clinical application of F-FLFA

To further assess its performance, 63 field samples, including 30 canine fecal samples, 18 oral and nasal mixed swab samples, and 15 blood samples, were tested using F-FLFA and then compared with RT-PCR for the detection of nonspecific amplification. As shown in Table 1, twenty of 30 fecal samples, 10 blood samples and 2 oral and nasal mixed swab samples were identified to be positive by F-FLFA, 29 of negative samples detected by F-FLFA were also negative for RT-PCR. However, only 32 of the 34 CDV-positive samples as determined by F-FLFA were also identified by RT-PCR. The amplicons from those two samples (blood samples) were purified and cloned into the pMD18-T vector for sequencing. Results demonstrated that the two samples were positive for CDV. The coincidence rate between RT-PCR and F-FLFA was 96.83% (Table 1). To determine the authenticity of the F-FLFA results, the PCR products of 32 positive samples were sequenced, revealing a 99% identity with the fusion gene of CDV.

**Table 1:** Coincidence rate of F-FLFA and RT-PCR.

		RT-PCR			CR
		Positive	Negative	Total	
F-FLFA	Positive	32	2	34	96.83%
	Negative	0	29	29	
	Total	32	31	63	

CR, Coincidence rate. CR = (32 + 29) / 63 × 100%.

## Discussion

CDV affects dogs and wildlife across various geographical regions, posing a considerable threat to both endangered and vulnerable species, such as Siberian tigers, Ethiopian wolves, red pandas, cheetahs, and lions (10, 14–17). Consequently, rapid and convenient diagnosis of CDV is crucial for clinical applications and the implementation of effective preventive measures. In the present study, we developed a rapid and sensitive assay (F-FLFA) for the successful detection of CDV.

Traditional diagnostic methods, with virus isolation regarded as the “gold standard” for CDV detection, utilize antigens, nucleic acids, and antibodies for clinical diagnosis in whole blood, serum, and cerebrospinal fluid (18–22). However, there is a demand for point-of-care diagnostic tests for CDV that can facilitate field diagnosis through isothermal amplification (23–25). FLFA analysis surpasses conventional tests in simplicity, enabling straightforward execution. Furthermore, FLFA based on the F and N proteins can detect antigens under visible light, thereby enabling preliminary diagnosis in pet hospitals (11, 26). To address the sensitivity constraints inherent in traditional LFA, previous research developed the F-FLFA approach utilizing fluorescent microspheres. These beads are distinguished by their exceptional magnetic properties, fluorescence, and capacity for biological modification, features that have found extensive application in the biomedical field (27–30).

In this study, we developed an effective assay (F-FLFA) for the detection of CDV based on the fluorescent-nanoparticle-labelled monoclonal antibodies. This assay offers considerable improvements over traditional detection methods, showing several advantages: (1) The assay showed excellent specificity and sensitivity, which has stability for antigen detection; (2) The assay required less than 5 min detection time, offering substantial time savings compared to the RT-PCR assay; (3) The assay required only mild reaction conditions, operating at room temperature with less complex equipment. Furthermore, according to the detection of clinical samples, it is demonstrated that F-FLFA is ideally suited for the rapid detection of clinical samples in veterinary clinics and field settings. There are potentially to improve the accuracy of detection. We hypothesized that a few unknown impurities affect the binding of antigens to antibodies. It is imperative for us to lyse the blood cells thoroughly or to optimize buffer composition.

Molecular diagnostic techniques, including polymerase chain reaction (PCR), quantitative PCR methods, loop-mediated isothermal amplification (LAMP), and recombinase polymerase amplification (RPA), have been developed and widely applied for pathogen diagnosis. However, these diagnostic methods can be impeded by a variety of reaction inhibitors, such as alkaline lysis, high genomic DNA concentrations, and lysis reactions, which can suppress enzymatic activity (31, 32). In contrast, F-FLFA allows for the direct testing of original samples, such as blood, nasal swabs, or culture media, without requiring nucleic acid purification, thus facilitating on-site detection. Moreover, the application of fluorescent labels in F-FLFA is anticipated to improve quantitative analysis performance (33). Our findings demonstrated that F-FLFA demonstrated high accuracy and reproducibility at various concentrations and correlated well with the RT-PCR in clinical samples, suggesting that this assay holds considerable promise for the development in point-of-care diagnosis.

In conclusion, we developed an effective assay (F-FLFA) utilizing fluorescent microspheres for detecting the fusion protein of CDV. Notably, the assay allowed for sensitive in detection of field samples and demonstrated no cross-reactivity with CPV, CCoV, CAV, FCV, FHV, or FPV. Thus, this assay provides an effective and rapid means for the clinical detection and field diagnosis of CDV infections.

## Data availability statement

The datasets presented in this study can be found in online repositories. The names of the repository/repositories and accession number(s) can be found in the article/Supplementary material.

## Author contributions

ZC: Writing – original draft, Writing – review & editing. LY: Writing – original draft, Writing – review & editing. XL: Writing – original draft, Writing – review & editing. JS: Writing – original draft, Writing – review & editing. YC: Writing – original draft, Writing – review & editing. EF: Writing – original draft, Writing – review & editing. XL: Writing – original draft, Writing – review & editing. YF: Writing – original draft, Writing – review & editing. XH: Writing – original draft, Writing – review & editing. WC: Writing – original draft, Writing – review & editing. FC: Writing – original draft, Writing – review & editing. SC: Writing – original draft, Writing – review & editing.

## Supplementary material

SUPPLEMENTARY FIGURE S1 F-FLFA performance **(A)** and readout of fluorescence intensity of test and control lines **(B)**.
