# Supplementary figures and images for: Rapid lateral flow immunoassay for fluorescence detection of canine distemper virus (CDV)
Source: Front Vet Sci. 2024 Jun 11;11:1413420. doi: 10.3389/fvets.2024.1413420 (PMC11197456; doi:10.3389/fvets.2024.1413420)

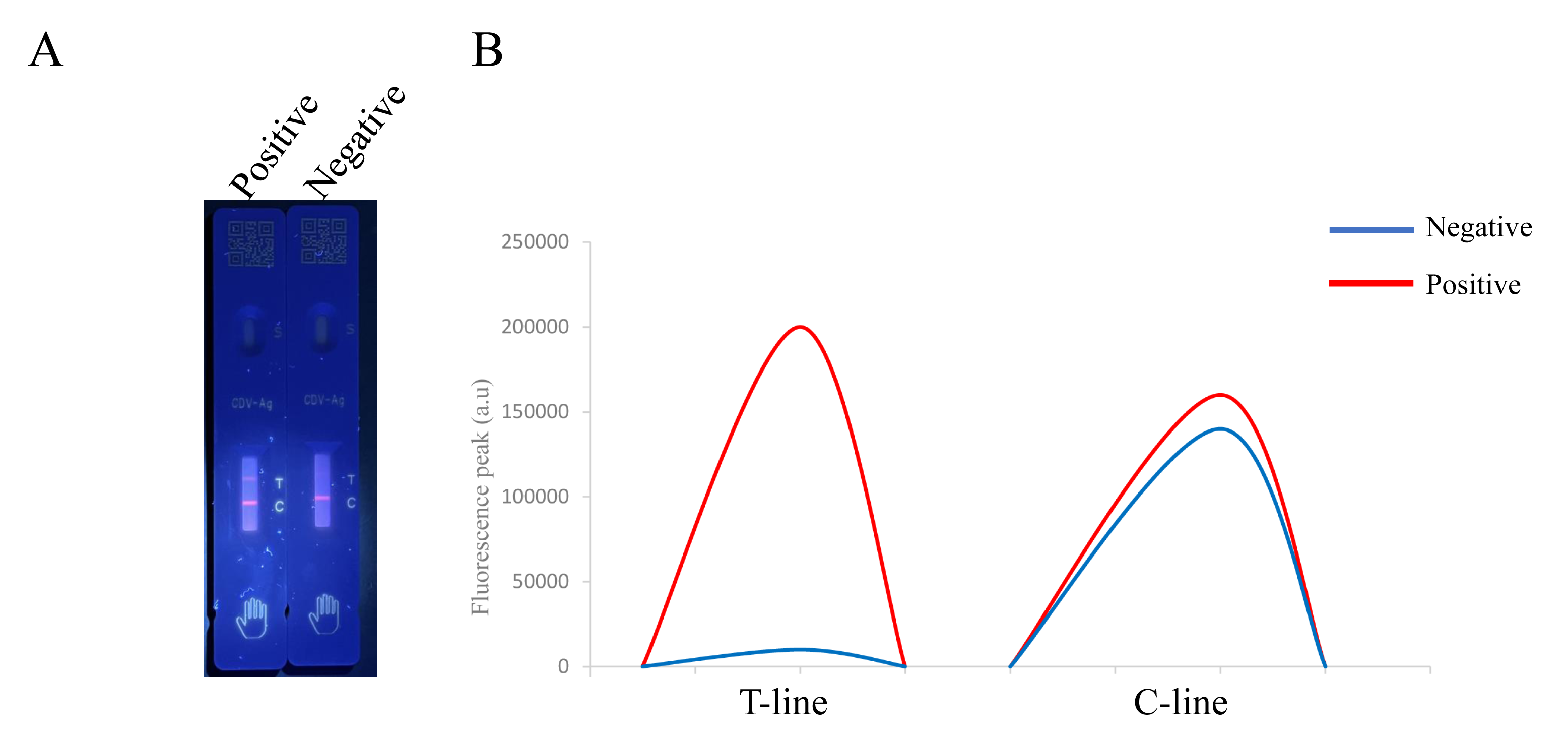

Supplement: SUPPLEMENTARY FIGURE S1 — F-FLFA performance (A) and readout of fluorescence intensity of test and control lines (B). [file Image_1.tif]
